# The Refraction of the Eye

**Published:** 1899-02

**Authors:** 


THE REFRACTION OF THE EYE. A Manual for Students. BY GUSTAVUS HARTRIDGE, F. R. C. S.; Senior Surgeon to the Royal Westminster Ophthalmic Hospital; Ophthalmic Surgeon and Lecturer on Ophthalmic Surgery to the Westminster Hospital, etc., etc. With 104 illustrations. Ninth edition. London: J. & A. Churchill, 7 Great Marlborough Street. 1898. Philadelphia: P. Blakiston's Son & Co.

This has been considered one of the standard works on refraction for a number of years in this country and England. The work is so well known it is unnecessary to go into extensive detail. The chapter on retinoscopy has been somewhat revised in this edition and the author quotes quite extensively from an American authority on the subject. In myopia he advises a stronger glass than most oculists care to prescribe, the majority agreeing (as he acknowledges) with Landolt that a full correction tends to increase the myopia by putting extra work upon the ciliary muscle. In aid to the clearness, cases are referred to, illustrating the point that it is desired to bring out. He speaks favorably of Tweedy's optometer, an instrument little known in this country. Optometers are not in favor here among ophthalmologists, as no instrument of the sort has been devised which has eliminated the factor of possible ciliary spasm. Muscle testing is dealt with superficially and this is all the more surprising as the author refers to prisms, decentering of lenses, and the like. In the appendix is a table of English and French inches with the equivalent in dioptrics, small type and large, for testing near and distant vision, also Pray's test types for astigmatism.

